# The International Collaboration of Pharmacy Journal Editors (ICPJE)formally constituted to foster quality around clinical and social pharmacy practice research publications^☆^

**DOI:** 10.1016/j.rcsop.2024.100519

**Published:** 2024-10-05

**Authors:** F. Alves da Costa, F. Fernandez-Llimos, S. Desselle, I. Arnet, Z. Babar, C. Bond, M. Cordina, V. Garcia Cardenas, M.S. El Hajj, R. Jacobsen, A.V. Law, L.S. Nørgaard, C. Polidori, N. Shcherbakova, D. Stewart, F. Tonin, A.E. Weidmann

**Affiliations:** a Research Institute for Medicines (iMed.ULisboa), Faculty of Pharmacy, University of Lisbon, Lisbon, Portugal; b International Journal of Clinical Pharmacy; c Laboratory of Pharmacology, Department of Drug Sciences, Faculty of Pharmacy, University of Porto, Porto, Portugal; d Revista Brasileira de Farmácia Hospitalar e Serviços de Saúde; e Touro University California College of Pharmacy, Vallejo, CA, USA; f Research in Social and Administrative Pharmacy; g Exploratory Research in Clinical and Social Pharmacy; h Department of Pharmaceutical Sciences, University of Basel, Basel, Switzerland; i University of Huddersfield, Huddersfield, UK; j Journal of Pharmaceutical Policy and Practice; k College of Pharmacy, QU Health, Qatar University, Doha, Qatar; l University of Aberdeen, Aberdeen, UK; m International Journal of Pharmacy Practice; n Department of Clinical Pharmacology and Therapeutics, WHO Collaborating Centre for Health Professionals Education and Research, University of Malta, Msida, Malta; o University of Granada Faculty of Pharmacy, Granada, Spain; p Department of Pharmacy, University of Copenhagen,Denmark; q Western University of Health Sciences, Pomona, CA, USA; r Experimental Medicine and Public Health, University of Camerino, Camerino, Italy; s European Journal of Hospital Pharmacy; t Western New England University, Springfield, Massachusetts, USA; u Faculty for Chemistry and Pharmacy, University of Innsbruck, Austria

The Granada statements were a result of the need to strengthen clinical, social and administrative pharmacy practiceas an area of knowledge that translates into practice, research and policy.As a response, a group of clinical and social pharmacy practice journal editors launched an initiative in Granada in 2022 to discuss ways to improve the quality of publications in this area, which culminated in the Granada statements. Eighteen statementswere developed, clustered into six main domains:1) the appropriate use of terminology; 2) developing impactful abstracts; 3) having the required peer reviews; 4) preventing journal scattering; 5) more effective and wiser use of journal and article performance metrics; and 6)authors' selection of the most appropriate pharmacy practice journal to submit their work. The full Granada statements have been published in 14 journals.^1^, ^2^, ^3^, ^4^, ^5^, ^6^, ^7^, ^8^, ^9^, ^10^, ^11^, ^12^, ^13^, ^14^ These pioneering statements are rooted in similar endeavors undertaken by scholars in other health professions groups, fostering the concept of interdisciplinary consensus and advancing scientific paradigm.^15^^,^^16^

Various chief editors, associated editors, and publishers met again in June 2024, this time in Basel, Switzerland. Following a consensus approach to select a name that best reflected the group scope, mission and vision, the name of the International Collaboration of Pharmacy Journal Editors (ICPJE) was selected. The ICPJE was then born.

During the meeting in Basel, the group discussed current issues relating to raising the quality of publications which among other things reflected a need to abate the discipline's need to re-evaluate itself though papers examining the importance of pharmacy by other stakeholders. These were not cited by papers outside or even within pharmacy, which has been demonstrated by a more holistic examination of drivers of citations through original research.^17^ The findings of this study highlight four main factors associated with citations, the paper's number of references, the year of publication, social media mentions and the topic area of research, namely outcomes associated with the actual provision of pharmacy services, and medication adherence (models and interventions more so than additional descriptive research). In the context of the discussion, it was emphasized that several previous studies across various disciplines including medical specialties, nursing and other allied healthcare professions have reported diverse findings. While some publications have concurred with the impact of social media highlighting its role in increasing visibility,^18^ most publications have found the nature of the topic and the methodology employed to be highly relevant factors.^19^ This corroborates withthe findings of Shcherbakova et al. and others who also pointed out the relevance of the journal reputation.^17^^,^^20^ Furthermore it was highlighted that the COVID-19 pandemic was found to boost citations.^20^ Another important determinant is innovation and multicentre or multidisciplinary studies. Some of the aforementioned studies have identified the number of references as a success factor; and this might be indicative of the paper having a more comprehensive a literature review and discussion.^17^^,^^18^

Additionally, the group did an analysis of the number of Medical Subject Headings (MeSH) used for indexation of clinical and social pharmacy practice articles compared to those in clinical medicine and similar areas and found a significantly lower number of terms. Furthermore, with full implementation of the automatic indexation by the National Library of Medicine in 2022, this problem was further exacerbated.^21^ It has been determined that an essential area that the ICPJE should focus their efforts on is to promote the standardization of terms used in pharmacy practice articles. This can be achieved, for instance, by promoting use of preferred terms to describe systems of care in pharmacy. It will then help focus searches by researchers and maximize the likelihood of important papers in pharmacy being located. Finally, the ICPJE editors will help prospective authors utilize MeSH terms in article titles and abstracts to coalesce our efforts in raising the visibility of pharmacy practice research and ameliorating ambiguity around terms not fully recognized by scholars, particularly those outside the discipline.

Whilst in Basel, those present reflected on the accessibility of the Granada statements if they were read without the underpinning justification included in the original paper. It was concluded that each statement needed to be accompanied by a few explanatory sentences, describing the underlying rationale and targeted at the audience for whom the statement was most relevant, i.e., publishers, editors, reviewers and most importantly authors. It was therefore considered crucial to include a wider audience in the revision of the statements and descriptions, embracing the concept of co-creation.^22^To achieve this, it was agreed that three subgroups should be convened. The first subgroup was tasked with composing short explanatory sentences to accompany each Granada statement. The second group was tasked with proposing a methodology to create an Early Career Researcher Advisory Board (ECRAB), defining their tasks and duties.The ECRAB will include authors and reviewers from different pharmacy practice journals and will act as a sounding board for the ICPJE, with an initial task to comment on short explanatory statements to accompany the Granada statements and eventually support any rewording needed. Similar initiatives have been proposed by the World Health Organization (WHO) Regional Office for Europe, with the creation of the Youth4Health special initiative, which aims to amplify and embed youth voices and perspectives into all areas of its work (https://www.who.int/europe/initiatives/youth4health). The ICPJE truly believes that this ECRAB has enormous potential to contribute to the external visibility and promotion of clinical and social pharmacy practice research paradigm. Finally, the third subgroup is focused on embedding the statements into university curricula and part of its duties will be creating a methodology to engage the Higher Education Institutions with the ultimate goal of increasing awareness about the statements and their influence and use starting at the undergraduate level. Even though the remit of the ICJPE expands way beyond Europe, the recent revision of the European Directive on minimum training requirements for pharmacists (and other healthcare workers)^23^ may be an excellent opportunity to ensure adequate knowledge and skills of scientific writing within the context of some of the new compulsory topics, such as pharmaceutical care, clinical pharmacy and public health, as a means to contribute to disseminate and promote knowledge and thus influence policy and practice.

In summary, the ICPJE was born from an initial small group that met previously in Granada to advance the visibility and quality of research in clinical, social and administrative pharmacy practice. Even prior to its formal naming, the group had made some progress in the past couple of years, although it recognizes the need to consolidate its work. The group is dedicated toward strengthening clinical, social and administrative pharmacy practice, not only as a discipline, but the entire profession, including the patients served by its clinicians and researchers. The ICPJE was founded by a select group of journals; but ultimately it is a group that is open to any other journal in the field. Each journal is represented by a vibrant group of individuals, including the editors and publishing companies. The ICPJE will be reaching out to various stakeholders seeking collaboration and insights from fellow scholars and practitioners throughout the world.

## CRediT authorship contribution statement

**F. Alves da Costa:** Conceptualization, Writing – original draft. **F. Fernandez-Llimos:** Conceptualization, Writing – review & editing. **S. Desselle:** Conceptualization, Writing – review & editing. **I. Arnet:** Writing – review & editing. **Z. Babar:** Conceptualization. **C. Bond:** Writing – review & editing. **M. Cordina:** Writing – review & editing. **V. Garcia Cardenas:** Writing – review & editing. **M.S. El Hajj:** Writing – review & editing. **R. Jacobsen:** Writing – review & editing. **A.V. Law:** Writing – review & editing. **L.S. Nørgaard:** Conceptualization, Writing – review & editing. **C. Polidori:** Writing – review & editing. **N. Shcherbakova:** Writing – review & editing. **D. Stewart:** Writing – review & editing. **F. Tonin:** Writing – review & editing. **A.E. Weidmann:** Writing – review & editing

## Declaration of competing interest

No conflicts of interest to declare.
